# Revealing the hidden signature of fault slip history in the morphology of degrading scarps

**DOI:** 10.1038/s41598-023-30772-z

**Published:** 2023-03-08

**Authors:** Regina Holtmann, Rodolphe Cattin, Martine Simoes, Philippe Steer

**Affiliations:** 1 CNRS, Institut de physique du globe de Paris, Université Paris Cité, 75005 Paris, France; 2 CNRS, Géosciences Montpellier, Université de Montpellier, 34000 Montpellier, France; 3 CNRS, Géosciences Rennes, Université de Rennes, 35000 Rennes, France

**Keywords:** Geomorphology, Tectonics, Natural hazards

## Abstract

Active faults accommodate tectonic plate motion through different slip modes, some stable and aseismic, others characterized by the occurrence of large earthquakes after long periods of inactivity. Although the slip mode estimation is of primary importance to improve seismic hazard assessment, this parameter inferred today from geodetic observations needs to be better constrained over many seismic cycles. From an analytical formulation developed for analyzing fault scarp formation and degradation in loosely consolidated material, we show that the final topographic shape generated by one earthquake rupture or by creep (i.e., continuous slip) deviates by as much as 10–20%, despite a similar cumulated slip and a constant diffusion coefficient. This result opens up the theoretical possibility of inverting, not only the cumulated slip or averaged slip rate, but also the number of earthquakes and their sizes from scarp morphologies. This approach is all the more relevant as the number of rupture events is limited. Estimating the fault slip history beyond a dozen earthquakes becomes very difficult as the effect of erosion on scarp morphology prevails. Our modeling also highlights the importance of trade-offs between fault slip history and diffusive processes. An identical topographic profile can be obtained either with a stable fault creep associated with rapid erosion, or a single earthquake rupture followed by slow erosion. These inferences, derived from the simplest possible diffusion model, are likely to be even more pronounced in nature.

## Introduction

**Figure 1:**
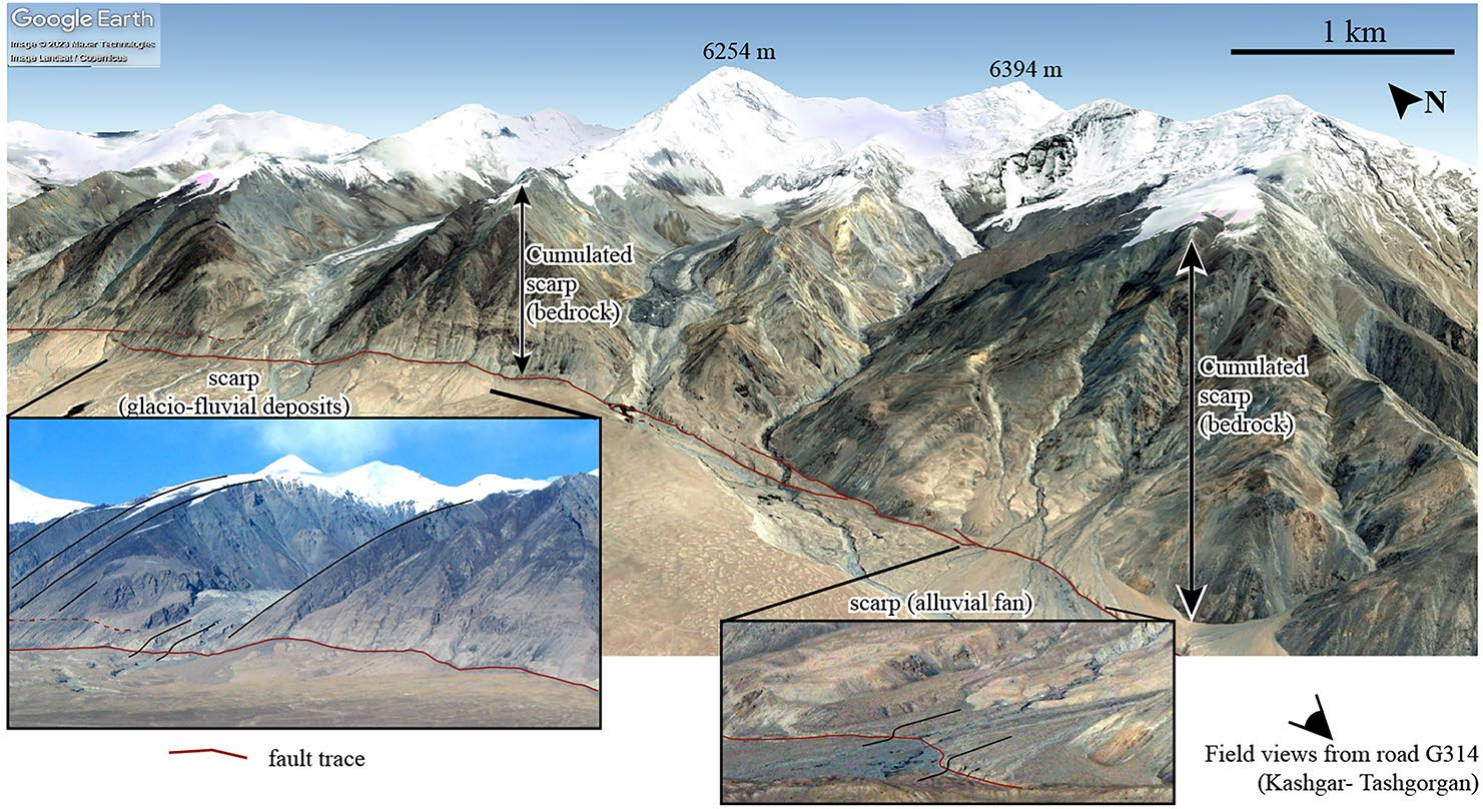
Normal fault scarp from the Kongur Shan extensional fault system^2,3^, using satellite and field views. A large (> 1000 m high) cumulated scarp, forming the mountain front, has exhumed bedrock over several million years. The overall slope of this long-term scarp decreases with altitude, as the exposure age of bedrock increases (left picture). Along strike, the normal fault disrupts more recent Quaternary deposits, such as glacio-fluvial sediments (lower left picture) or alluvial fans (lower right picture) and forms more subtle (1–20 m high) scarps. Erosion of scarps by diffusion, as considered hereafter in this study, only applies to these most recent features in loosely non-consolidated materials. Satellite general view from © Google Earth (2023 Maxar Technologies and Landsat/Copernicus), dating from July 9th 2007. Field views taken on October 18th 2007, along the road G314 from Kashgar to Tashgorgan (38° 43′ 42.97″ N, 75° 0′ 59.51″ E). The contrast of the original field pictures has been increased using Adobe Photoshop for an easier visibility of the landscape features. The original non-interpreted satellite view and field pictures are provided in supplementary material (Figs. S3 to S5). Figure generated using Adobe Illustrator.

Scarps are landscape features forming a steep break in the local or regional topographic slope. They relate to fluvial incision (i.e., terrace risers) or faulting (i.e., fault scarps) and can be of different sizes, from ca. 1–10 m high scarps for terrace risers or recent seismic scarps, and up to > 100 m high scarps for mature faults. Scarps result from a relative vertical movement (river incision in the case of terrace risers, tectonic uplift in the case of fault scarps). They are degraded by surface processes, leading to a decrease in their steepness over time. This latter observation provided the long-lived basis for the relative dating of these landscape features^1^ and later for various attempts at quantifying their age. An illustration is provided from field and satellite views of the Kongur Shan extensional fault system^2,3^ (Fig. 1). Bedrock has been exhumed along the large (> 1000 m high) cumulated fault scarp that forms the mountain front. The overall slope of this cumulated scarp decreases with altitude, in line with the progressively increasing age of bedrock exposure and exhumation^3^ (Fig. 1, left picture). Along strike, the fault trace is marked by more subtle (1–20 m high) and younger scarps that disrupt recent deposits (Fig. 1, right and left pictures). Based on their offset and age, these scarps have been used to quantify Quaternary fault slip rates^2^. Among these, the older higher scarp disrupting glacio-fluvial deposits (Fig. 1, left) appears less steep than the younger and smaller one within a more recent alluvial fan (Fig. 1, right). Altogether, these various features are clear indications that the fault is active. However, their simple inspection does not permit at the moment to assess how fault slip is accommodated over time, during earthquakes or by creep.

In loosely consolidated material (e.g., fluvial deposits, alluvial fans—as in the case of the most recent scarps in Fig. 1), slope-dependent processes favor the erosion of the higher and steeper parts of a scarp, while deposition of the eroded material occurs downhill, with a flux of sediment overall proportional to the local slope. These processes can be modeled by the progressive diffusion of a steep topographic slope^4,5^, providing from there a conceptual framework to constrain the diffusion age $\kappa t$ of such scarps and, in turn, infer their age $t$ or their coefficient of diffusion $\kappa$. This approach has been applied for the dating of terrace risers and associated terrace abandonment and incision^6–8^ or to provide chronological constraints on seismic fault scarps assuming that they formed after one single earthquake^1,5,9,10^, even though some complexities to these various approaches on the analysis of diffused scarps have been pointed out: some studies^9^ advocate for a decrease in the diffusion coefficient $\kappa$ with time to reconcile the morphology of young (high $\kappa$) and old (low $\kappa$) fault scarps and climate warming and the shift from glacial to inter-glacial are generally suggested to explain possible variations^10^.

Interestingly, in the specific case of fault scarps, most do not result from a single earthquake rupture but from several consecutive ruptures or even creep. Multiple fault ruptures or creep will rejuvenate the overall fault scarp and maintain it steep (or relatively steeper) over time^5,6,9^. This latter point raises the possibility that there could be a hidden morphological signature of whether a fault scarp was built by one or multiple earthquake ruptures or by creep. Active faulting leaves a morphological signature (Fig. 1) that has been amply used in numerous studies to quantify active tectonics^11^. However, whether landscapes keep a record of how active faults slip, either seismically or aseismically, has remained largely unexplored^12,13^. With the increasing availability of high-resolution topographic data (< 1 m resolution), there is a timely opportunity to investigate this issue^14,15^.

Here, we aim to illuminate fundamental aspects of the influence of tectonic slip history, seismic or aseismic, on the morphology of degrading fault scarps. We investigate and explore this idea by considering the simple case of scarps in non-cohesive materials, such as those disrupting alluvial fans or fluvial deposits (see recent scarps pointed out in Fig. 1), where diffusion-like erosion is the primary process degrading the scarps. We first develop a new analytical formulation for scarp evolution, including fault slip by earthquake ruptures or by creep, coupled to linear topographic diffusion, which is considered as the simplest model of scarp degradation^5,8,16^. We then systematically investigate the morphological signature of fault slip history, including the role of the number of ruptures, the coefficient of diffusion, and the time since the onset of fault activity. Last, we revisit with this model the time dependency of diffusion coefficient associated with a previously documented field case in Central Greece to reveal the potential role of fault slip history in scarp morphology.

## Method

### Fault uplift and scarp degradation model

In our approach, for the sake of simplicity, we consider a very steep fault (${\text{dip angle}}\to 90^\circ$) for which we neglect the effect of horizontal tectonic advection. Hence, the elevation profile $z$ across the scarp is initially:

1 $$z\left(x<0, t=0\right)=0 \quad and \quad z\left(x\ge 0, t=0\right)=u,$$

with $x$ the horizontal distance from the fault trace, positive towards the uplifting block (hanging-wall for a reverse fault, footwall in the case of a normal fault), $t$ the time, and $u$ the uplift leading to the height of the initial scarp.

We consider different fault slip and uplift scenarios, which all reach the same final total uplift: (1) a fault scarp formed during one single earthquake rupture; (2) a fault scarp resulting from multiple identical and periodical earthquake ruptures; and (3) a fault scarp formed by continuous slip on a creeping fault. In all scenarios, the fault scarp is subjected to surface processes, modeled here using the well-established linear diffusion model^5,8,16^:

2 $$\frac{\partial z}{\partial t}=\kappa \frac{{\partial }^{2 }z}{\partial {x}^{2}},$$

where $\kappa$ is a diffusion coefficient, assumed here constant through space and time. This model is generally considered relevant to describe erosion of loosely unconsolidated rocks subjected to creep, bioturbation and rainsplash on low-gradient slopes.

### Analytical solution for a single fault uplift

The solution to Eq. (2) for the degradation of a topographic step related to a single vertical rupture, as defined by the initial conditions in Eq. (1), is^5^:

3 $$z\left( {x,t} \right) = u \times {{\left[ {1 + {\text{erf}}\left( {\frac{x}{{\sqrt {4\kappa t} }}} \right)} \right]} \mathord{\left/ {\vphantom {{\left[ {1 + {\text{erf}}\left( {\frac{x}{{\sqrt {4\kappa t} }}} \right)} \right]} 2}} \right. \kern-\nulldelimiterspace} 2}$$

where $u$ is the height of the rupture, $t$ is the time elapsed since the rupture, and erf is the error function defined as

4 $${\text{e}}{\text{r}}{\text{f}}\left(\eta \right)=\frac{2}{\sqrt{\pi }}{\int }_{0}^{\eta }{e}^{-{\gamma }^{2}}d\gamma .$$

**Figure 2:**
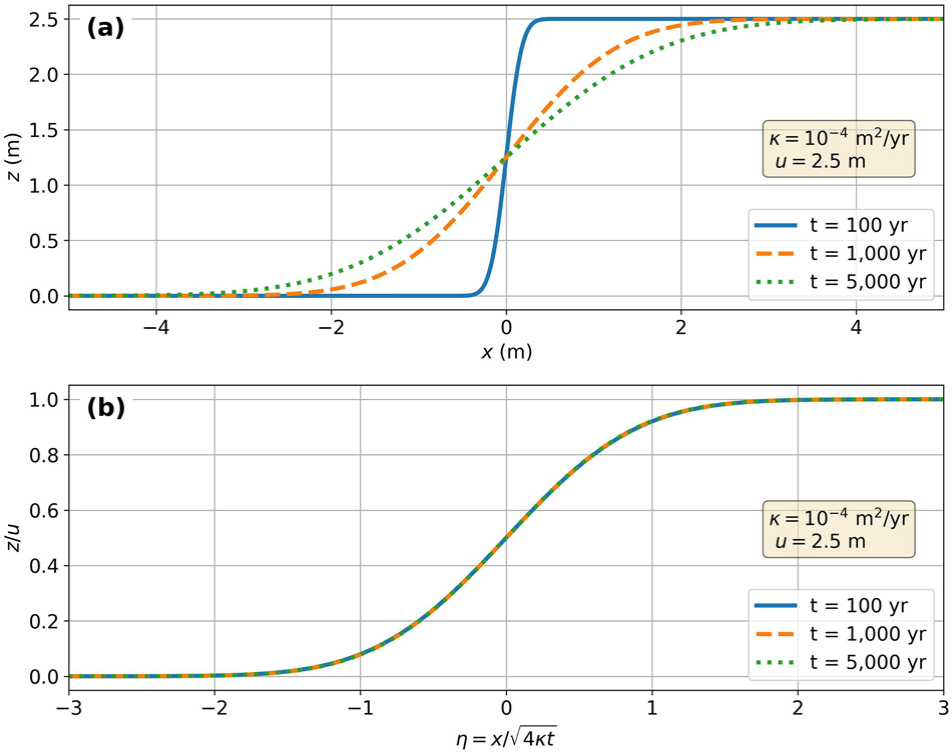
Evolution of a degrading fault scarp. (**a**) Elevation profiles $z\left(x\right)$ across a fault scarp assuming a single rupture uplift $u=2.5$ m and a diffusion coefficient $\upkappa ={10}^{-4}$ m^2^/yrea. Blue, orange, and green lines show the degraded scarp profiles after 100, 5000, and 10,000 years, respectively. (**b**) Same profiles using the dimensionless parameters $\eta$ and $z/u$. Figure generated using the Python’s matplotlib library.

Figure 2a illustrates the diffusion of a fault scarp formed after one earthquake rupture (i.e., one uplift event). Erosion and sedimentation modify rapidly and significantly the topography near the fault. Over time, as the scarp profile degrades and flattens, the area affected by surface processes widens, and the scarp degradation rate decreases.

Equation (3) can be expressed in a dimensionless form with the dimensionless parameters $z/u$ and $\eta =x/\sqrt{4\kappa t}$

5 $$\frac{z\left(x,t\right)}{u}=\left[1+erf\left(\eta \right)\right]/2.$$

The resulting dimensionless diffusion profile across the scarp is independent of both the uplift magnitude and the time elapsed since the uplift event (Fig. 2b). In the case of a single earthquake rupture, the final topographic profile that diffused over a total time-lapse ${t}_{end}$ can be calculated using the initial (and total) vertical offset as $={u}_{\mathrm{end}}$ :

6 $$\frac{z\left(x, {t}_{end}\right)}{{u}_{end}}=\left[1+{\text{erf}}\left({\text{x}}/\sqrt{4\kappa{\text{t}}_{\mathrm{end}}}\right)\right]/{2}.$$

### Analytical solution for multiple fault uplifts or creep

As the considered diffusion degradation is uni-dimensional and linear, the scarp profiles resulting from multiple events can be constructed through a combination of diffused incremental topographies. The case of a double-uplift scenario, with diffusion during a total duration of ${t}_{end}$, is illustrative (See Supplementary information S1 and Fig. S1). Two uplift events, each with a vertical offset of ${u}_{\mathrm{end}}/2$ occur at $t=0$ and $t={t}_{end}/2$. The final topographic profile results from the sum of two diffused incremental topographies: one, associated with the first event at $t=0$, diffuses throughout the whole model duration ${t}_{end}$, whereas the other, related to the second event at $t={t}_{end}/2$, diffuses during a time-lapse of ${t}_{end}/2$ only. The initial topographies being similar (i.e., a Heaviside step function with a vertical offset of ${u}_{end}/2$), the formulation for the resulting profile is:

7 $$\frac{z\left(x, {t}_{end}\right)}{{u}_{end}}=\frac{1}{{2}}\left(\left[1+{\text{erf}}\left({\text{x}}/\sqrt{4\kappa{\text{t}}_{\mathrm{end}}/2}\right)\right]/2 + \left[1 + {\text{erf}}\left({\text{x}}/\sqrt{4\kappa{\text{t}}_{\mathrm{end}}}\right)\right]/{2}\right).$$

**Figure 3:**
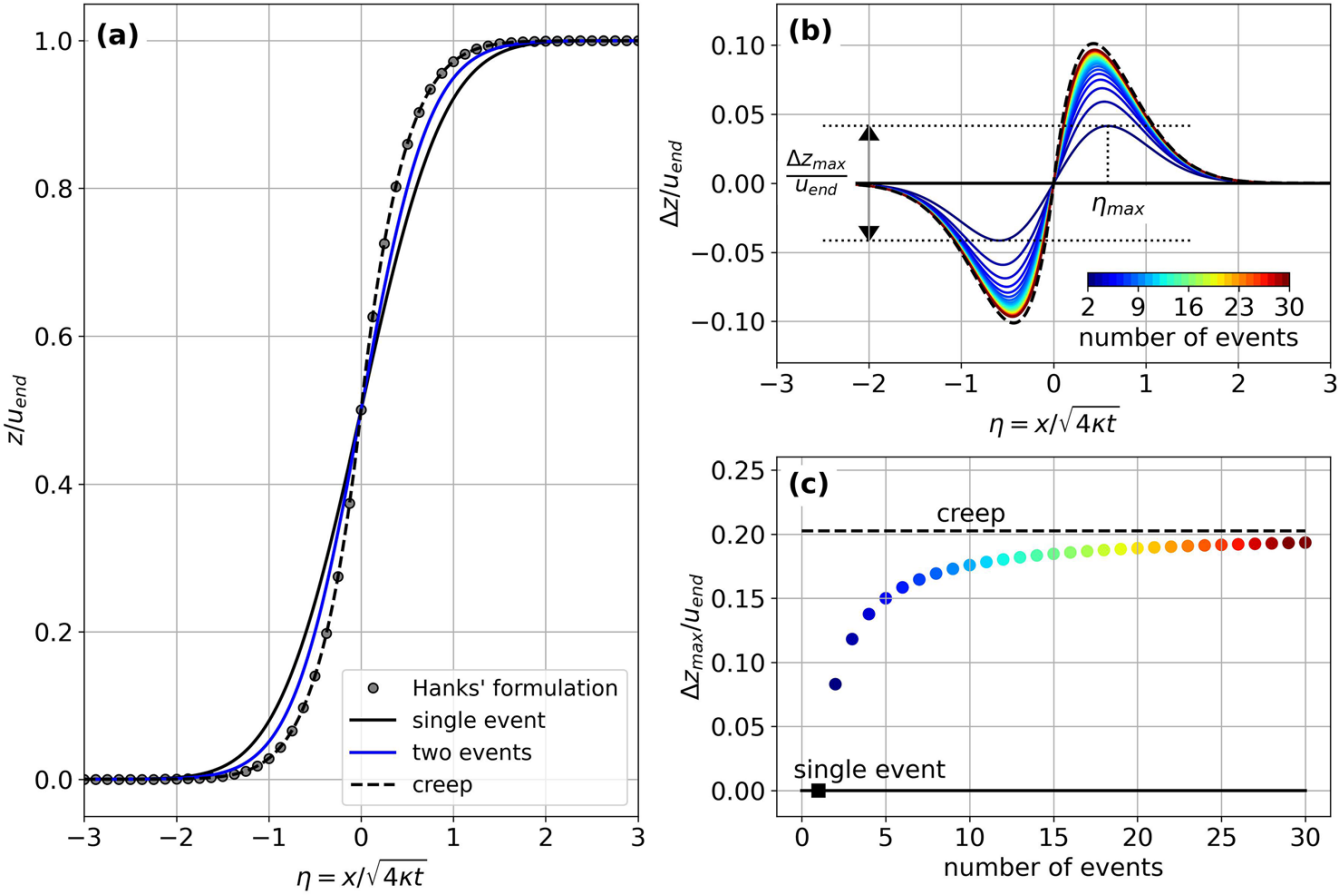
Influence of the number of rupture events on scarp profiles associated with a total uplift of ${u}_{end}$ and a final time-lapse of ${t}_{end}$. The thick and dashed black lines give the result for the single-event scenario and a creeping fault, respectively. The color scale is related to the number of events. (**a**) Calculated dimensionless profiles. Hanks’ formulation^5^ is given in Supplementary information S2. (**b**) Topographic deviation with respect to the single-event scenario. (**c**) Variation of the maximum deviation with the number of uplift events. Figure generated using the Python’s matplotlib library.

By extension, the degradation of a scarp formed during a multiple-uplift scenario, with periodical ruptures, is given by:

8 $$\frac{z\left(x,{t}_{end}\right)}{{u}_{end}}=\frac{1}{2\times n}{\sum }_{i=1}^{n}\left[1+{\text{erf}}\left(x/\sqrt{4\kappa {t}_{end}\times \frac{i}{n}}\right)\right],$$

with $n$ the number of uplift events. In the case of a continuous uplift scenario (i.e., creep), $n$ tends to infinity, and each event generates a vertical uplift of ${u}_{end}/n$. Here, we simulate the creeping behavior using Eq. (8) with $n={10}^{4}$. This formulation gives similar results to those obtained from Eq. (13) of Hanks^5^ (Fig. 3a), in which the scarp morphology of a creeping fault is the solution of the equation

9 $$\frac{\partial z}{\partial t}=\kappa \frac{{\partial }^{2}z}{\partial {x}^{2}}+A,$$

where $A={u}_{end}/{t}_{end}$ is the uplift rate (see supplementary material S2 for mathematical formulations).

## Results

### Effect of uplift scenarios

Using Eq. (8), we first calculate the diffusion profiles associated with the degradation of a scarp generated by *n* total identical events, with $n$ ranging from 1 (single rupture) to ${10}^{4}$ (creep) (Fig. 3). Although the cumulative uplift equals ${u}_{end}$ at the final time ${t}_{end}$ in all scenarios, each uplift scenario produces a topographic profile that significantly differs from the others (Fig. 3a). The greater the number of events, the steeper the slope near the fault.

Compared to the single uplift scenario, the deviation between the resulting diffusion profiles is maximal at the dimensionless horizontal distance of $\eta =\pm {\eta }_{max}$ from the fault (Fig. 3b). The peak-to-peak deviation reaches up to ca. 20% of ${u}_{end}$ for the creep scenario. The greater the number *n* of uplift events, the smaller the difference in deviation associated with an additional uplift (Fig. 3b,c). For example, this deviation is ca. 8%, 16%, 18%, and 19% of ${u}_{end}$ when *n* equals 2, 6, 12, and 22, respectively. The parameter ${\eta }_{max}$ is relatively constant, slightly decreasing from ca. 0.6 to ca. 0.4 when increasing $n$ (see Supplementary material S3).

A maximum deviation of a few percent may at first seem insignificant. However, rates of erosion or sedimentation by diffusion vary significantly with time. They are fast at the beginning due to greater slopes and slow down with the decrease of the topographic curvature^6^. Therefore, the observed deviation, even though modest, could significantly impact the estimate of parameter $\kappa t$ and, from there, the inferred diffusion coefficient or the age of a scarp.

### Synthetic tests

We examine the potential to identify an uplift history (i.e., single or multiple earthquake ruptures, or creep) by analyzing a given scarp profile. This approach compares a well-defined synthetic profile to profiles produced from various scenarios by varying $n, \kappa t$, and topographic noise.

First, we consider a reference model obtained with ${n}^{ref}=5$ ruptures and $\kappa {t}^{ref}=1$ m^2^. A normal distributed random noise is added to this profile with a mean of zero and a standard deviation of ${\upsigma }^{ref}={10}^{-3}\times {u}_{end}$. This noise accounts for the accuracy of topographic data and the natural heterogeneity and variability of scarp profiles. Next, we perform sensitivity tests assuming “true” models similar to the reference model, except for the tested parameter (either $n$, $\upkappa t$, or $\upsigma$). Finally, we perform the inversion by systematically exploring the model parameters with $1\le n\le 20$ and $0.1 \le\upkappa t\le 10$ m^2^. We calculate for each tested profile its likelihood $L$ relative to the “true” profile, defined as

10 $$L\left(n,\kappa t\right)=exp\left(-\frac{1}{N}{\sum }_{i=1}^{N}{\left[\frac{{z}_{i}-{z}_{i}^{true}}{{\sigma }_{i}^{true}}\right]}^{2}\right)$$

with $N$ the amount of data along the resulting profiles ($N =5000$ in our approach), ${z}_{i}$ the topographic profile associated with $n$ and $\kappa t$, ${z}_{i}^{true}$ the “true” profile, and ${\sigma }_{i}^{true}$ the noise. The likelihood distribution is normalized to the likelihood of the best-fitting profile to compare the different tests. The normalized likelihood ranges from 0 (poor agreement with the “true” profile) to 1 (good agreement).

**Figure 4:**
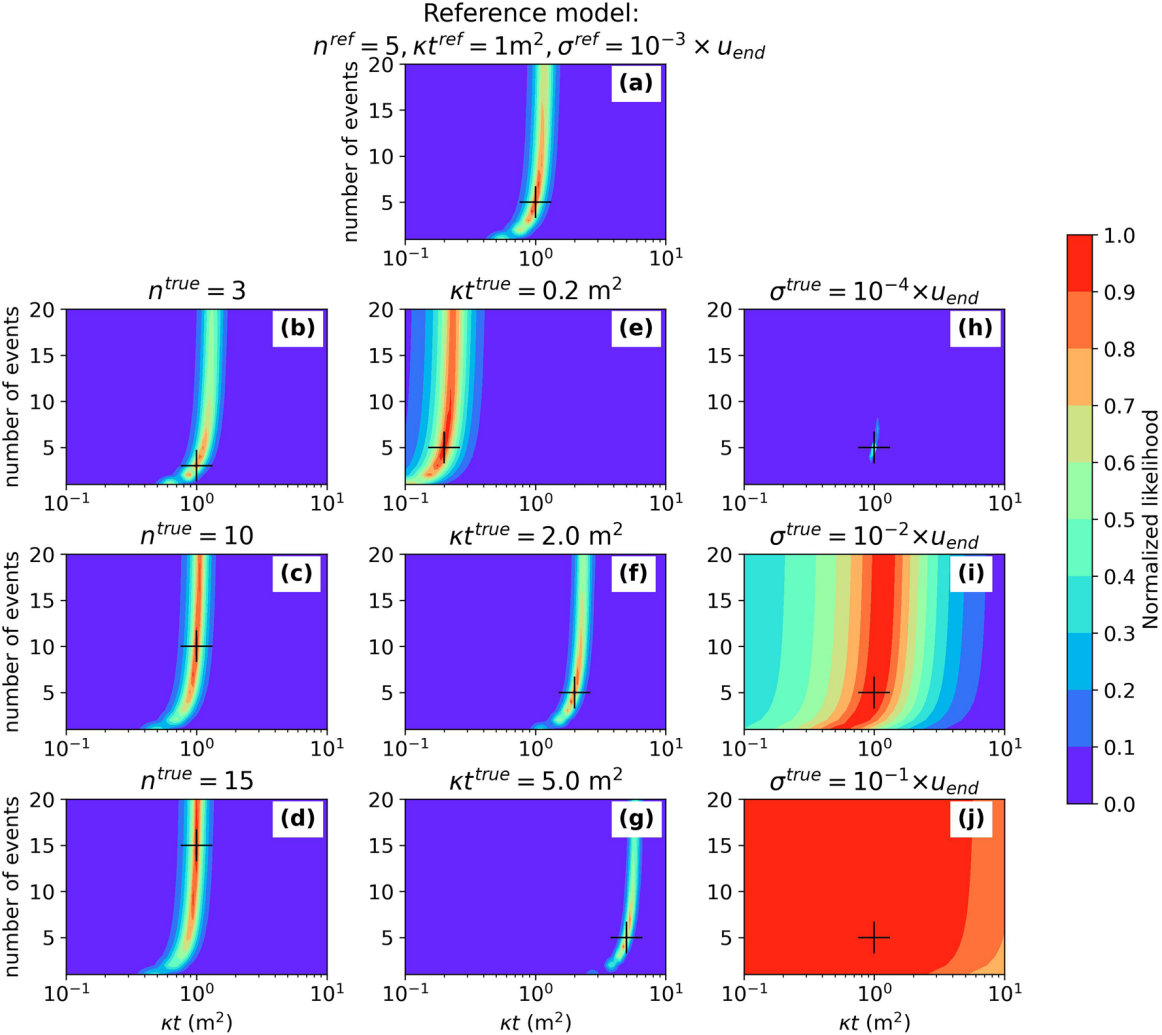
Likelihood distribution associated with synthetic tests. The black cross corresponds to the “true” model parameters. (**a**) Reference model associated with a five-events scenario, a constant $\upkappa t = 1$ m^2^, and a noise level of ${10}^{-3}\times {u}_{end}$. (**b**–**d**) Effect of the number of events with ${n}^{true}$ ranging from 3 to 15, $\upkappa {t}^{true}=\upkappa {t}^{ref}$ and ${\upsigma }^{true}={\upsigma }^{ref}$. (**e**–**g**) Influence of the $\upkappa t$ parameter, with $\upkappa {t}^{true}$ varying from 0.2 to 5 m^2^, ${n}^{true}={n}^{ref}$ and ${\upsigma }^{true}={\upsigma }^{ref}$. (**h**–**j**) Sensitivity to the topographic noise, with noise level between ${10}^{-4}\times {u}_{end}$ and ${10}^{-1}\times {u}_{end}, { n}^{true}={n}^{ref}$ and $\upkappa {t}^{true}=\upkappa {t}^{ref}$. Figure generated using the Python’s matplotlib library.

For the reference model, we obtain good normalized likelihoods ($L>0.9$) for $0.4 \le \kappa t \le 1.6$ m^2^ and $4 \le n \le 7$ (Fig. 4a), similar to the values of reference parameters. The obtained likelihood distribution also suggests a strong trade-off between $\kappa t$ and $n$ for models with $n<10$. Beyond this threshold, the likelihood only depends on $\kappa t$. This result is consistent with our previous observation that the diffusion profile is only weakly affected by the number of events for $n >10$(Fig. 3b,c). Additionally, the likelihood of a profile resulting from one or many ruptures can be similar if $\kappa t$ is adjusted (Fig. 4a). This result confirms that even though the effect of $n$ on the resulting profiles remains modest (with a difference of only a few percent; Fig. 3), assuming a scarp formed after a single rupture—instead of 5—can lead to underestimating $\kappa t$ by a factor of two.

We test the influence of changing the number of earthquake ruptures on the likelihood distribution, with $${n}^{true}$$ equal to 3, 5, 10, and 15, while keeping $${\kappa t}^{true}=1$$ m^2^ and $${\sigma }^{true}={10}^{-3} \times {u}_{end}$$ (Fig. 4b–d). In each case, the best likelihood is obtained for—or close to—the “true” values of the parameters. Our results suggest that the larger the parameter $${n}^{true}$$, the more difficult it is to retrieve this parameter from our inversions, and the easier it is to constrain $${\kappa t}^{true}$$. Indeed, a larger $${n}^{true}$$ leads to a wider zone with a good likelihood ($$L>0.9)$$ in the parameter space.

We also test the influence of varying ${\kappa t}^{true}$, between 0.2 and 5 m^2^, while keeping ${n}^{true}=5$ and ${\sigma }^{true}={10}^{-3} \times {u}_{end}$ (Fig. 4e–g). In each case, the best likelihood is obtained for—or close to—the “true” parameter values. Yet, we observe a wider zone associated with good likelihood, in the parameter space, for low values of ${\kappa t}^{true}$. Therefore, the inversion is more likely to confidently retrieve model parameters for large ${\kappa t}^{true}$, when diffusion has significantly modified the scarp morphology (i.e., old scarps or high diffusion rates).

To assess the impact of topographic noise on our inversion results, we perform modeling with ${\sigma }^{true}$ ranging between ${10}^{-4} \times {u}_{end}$ and ${10}^{-1} \times {u}_{end}$ (Fig. 4h–j). Regardless of the noise level, the best likelihood is obtained for—or close to—the “true” parameter values. Our results un-surprisingly show that noise affects the estimated range of good likelihood around the best-fit parameters, with a lower noise level leading to a narrower and better-defined range. Parameter ${\sigma }^{true}={10}^{-1} \times {u}_{end}$ gives a mostly uniform likelihood, suggesting that the approach presented here cannot be applied to the analysis of noisy topographic profiles (i.e., low data accuracy or large along-scarp variability).

### Application to the Eliki fault scarp (Greece)

**Figure 5:**
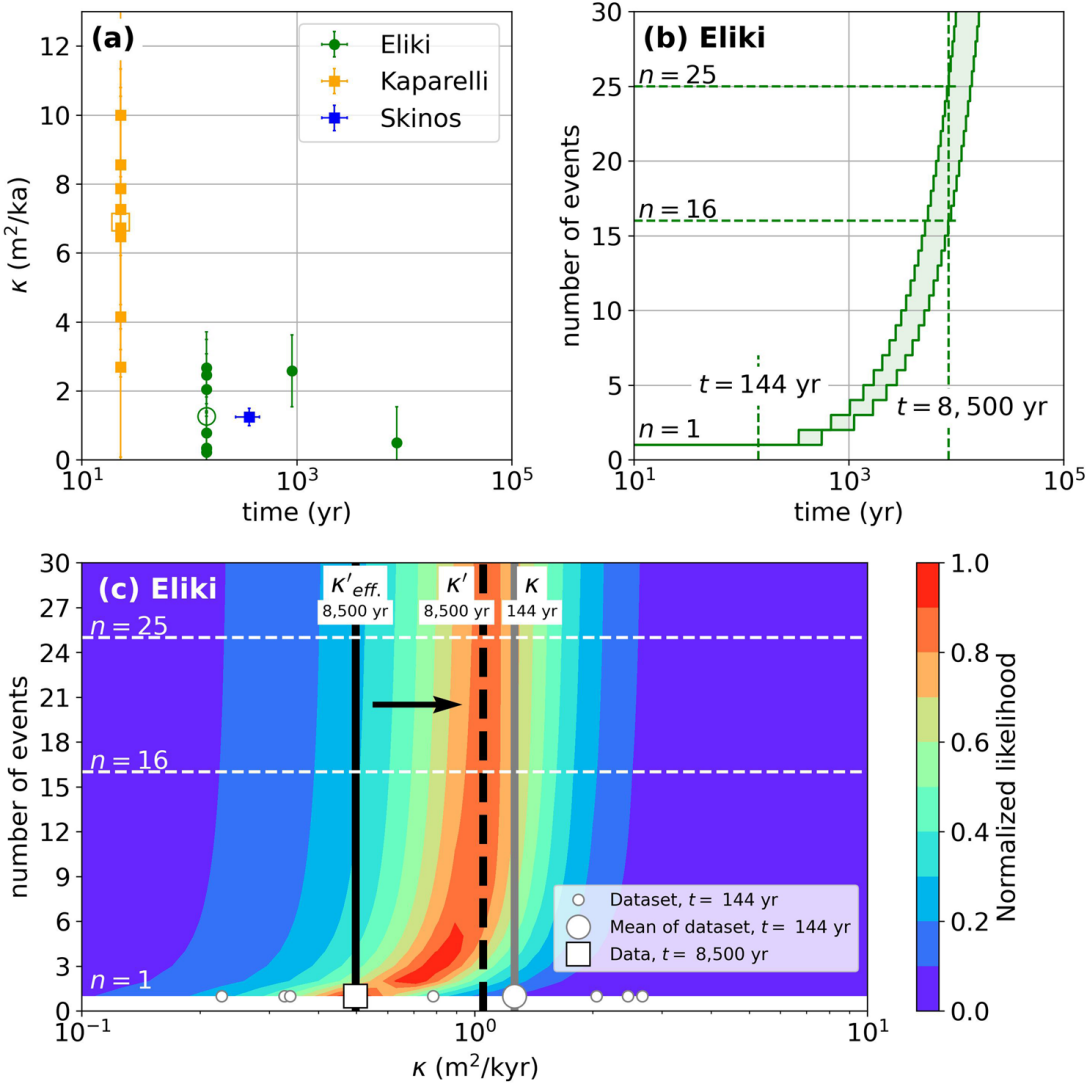
Influence of the number of events on the diffusivity coefficient $\upkappa$ as inferred from measurements along active faults in Greece. (**a**) Time variation of $\upkappa$ along the Eliki, Kaparelli, and Skinos faults, as proposed by Kokkalas and Koukouvelas^9^. (**b**) Relationship between the number of events and the degradation time along the Eliki fault^9^. (**c**) Likelihood of scarp profiles along the Eliki fault, assuming a single-event scarp that degraded during 8500 years with ${\upkappa }_{eff.}^{\mathrm{^{\prime}}}\sim 0.5$ m^2^/kyr as the “true model”. The vertical black line and the white square give the effective diffusivity determined in a trench, assuming a single event scenario^9^. The thick vertical dashed line is associated with κ′ = 1.05 m^2^/kyr as re-assessed from the calculated likelihood distribution and taking into account the 16–25 events that potentially ruptured this fault over the last 8500 years. Small circles give the diffusivity from field measurements on the fault scarp formed after the 1861 Eliki earthquake. These individual values yield a mean diffusivity of 1.26 m^2^/kyr for a duration of t = 144 years (large circle and vertical gray line). Figure generated using the Python’s matplotlib library.

The Gulf of Corinth (Central Greece) is one of the most seismically active regions in Europe^17^. Its rapid extension rate of 11–15 mm/year is accommodated along active normal faults bounding the rift^18–20^. Kokkalas and Koukouvelas^9^ investigated the degradation of normal fault scarps formed after the 1861 (Eliki) and 1981 (Kaparelli) earthquakes, which affected the southern and eastern regions of the Gulf of Corinth (Fig. S2). By considering that the scarps degraded by linear diffusion after these earthquakes, they determined the diffusion coefficient $\kappa$ that best fitted the observed morphologies and applied a similar approach to older scarps re-interpreted from paleoseismological trenches. By combining these results with the ages of the scarps, they propose that $\kappa$ decreases exponentially with time (Fig. 5a). Interestingly, these authors noted that the $\kappa$ of composite scarps (i.e., recording multiple ruptures over a longer time) was lower when considering the overall scarp rather than only the most recent segment formed during the last earthquake, in line with the documented decrease of $\kappa$ with time. They also note that these composite scarps can be erroneously modeled as if they formed during a single earthquake.

Here, we apply our multiple-rupture approach to test the impact of this simplification. To avoid lithological effects that may affect $\kappa$, we focus on the results from the Eliki fault. This fault system is located in the southwestern Gulf of Corinth. It consists of two north-dipping fault segments, both of ca. 15–20 km in length (Fig. S2). The Eliki sites studied by Kokkalas and Koukouvelas^9^ are located along the eastern segment. The investigated scarps are formed within poorly indurated Plio-Quaternary syn-rift fan delta conglomerates and alluvial gravels, and are therefore consistent with our modeling framework. We do not re-analyze the observations and processing performed on trench logs and scarps profiles in the following. Instead, we consider the mean diffusion coefficient $\kappa \sim 1.3$ m^2^/kyr derived for the 1861 scarp and the one $\kappa^{\prime}_{eff}\sim 0.5$ m^2^/kyr obtained in the T13 trench and covering a time-lapse of ca. 8500 years, as derived and determined by Kokkalas and Koukouvelas^9^. Based on the estimated recurrence interval of 450 ± 109 years, these diffusion coefficients can be related to a single event and to 16–25 events, respectively (Fig. 5b).

We consider the diffusion profile associated with a single-earthquake scenario, ${\upkappa }^{true}=$ 0.5 m^2^/kyr (old scarp), and ${\sigma }^{true}={10}^{-2} \times {u}_{end}$. We then calculate the likelihood of profiles associated with 1 ≤ *n* ≤ 30 and 10^−1^ ≤ *κ*′ ≤ 10^1^ m^2^/kyr. In the case of *n* = 16–25, we obtain good likelihoods ($L>0.9$) for 0.89 ≤ *κ*′ ≤ 1.17 m^2^/kyr, i.e., for *κ*′ similar to the average $\upkappa$ estimated from the recent 1861 scarp (Fig. 5c). A diffusion parameter of 1–1.2 m^2^/kr constant over time can therefore explain the diffusion profiles of both recent single-event scarps and multiple-event scarps in Eliki. This result suggests that the deduced decrease of the diffusion coefficient with time might be apparent and instead reflect the influence of tectonic slip history on the morphology and shape of the composite scarp.

## Discussion

Our results rely on the simplest model of fault scarp evolution, with a block uplift by slip on a vertical fault and degradation by linear diffusion. Even though linear diffusion has been successfully applied to specific field settings^21^, there is ample evidence that hillslope and scarp diffusion may not be linear^22,23^. Mass wasting and non-local sediment transport processes are also ignored while likely playing a significant role after each earthquake rupture. Additionally, we consider only an initial flat topography, even though the contribution of initial topography can be added to our mathematical formulation^6,24^. Given these simplifications, our modeling should not be considered as a faithful description of reality. However, it should be taken as a helpful way to isolate the specific impact of fault slip history on scarp morphology. We recall here that erosion by diffusive processes is only applicable where scarps disrupt loose and unconsolidated material such as alluvial fans or fluvial deposits.

Under these limitations, our results demonstrate that the slip history of a fault, whether it creeps or slips during one or several earthquake ruptures, leaves a potentially detectable morphological signature. For the same diffusion coefficient $\kappa$ and cumulated slip ${u}_{end}$, a creeping fault will lead to a steeper scarp than the one formed after a single earthquake rupture, with up to 20% of topographic deviation. In turn, this result implies that it is theoretically possible to invert the diffusion age and the number of uplift events that formed a scarp; however, only when this number is reduced ($<$ 10 uplift events), otherwise only the diffusion age can be robustly inferred. These inferences can only be achieved for a limited topographic noise, significantly lower than ca. 10% of the cumulated scarp height. More specifically, topographic noise needs to be significantly lower than the maximum deviation expected between topographic profiles resulting from different slip scenarios – i.e., $\ll 20\%$ of the total scarp height (Fig. 3b,c), otherwise the morphological signature of fault slip history remains hidden and undetectable.

The model developed in this paper succeeds in reconciling the apparent paradigm^9^ of young and old scarps associated with high and low apparent $\kappa$, respectively, by accounting for their contrasting slip history. We note that, due to the principle of superposition, a linear model of diffusion should lead to the least obvious effect of fault slip history on scarp morphology. This effect is, therefore, likely to be enhanced when considering more complex and potentially non-linear models. Since high-resolution (< 1 m) topographic data are now becoming extensively available^25^, our study will help revisit the age-morphology of scarps and, more interestingly, help better constrain the slip history of active faults. Our results imply that there could be more to be extracted from the morphology of landscapes than just the rates of active faulting, as extensively done in numerous studies. Indeed, the kinematics of faulting (seismic ruptures vs. creep) may leave a subtle signature yet to be fully explored.

## Supplementary Information

Supplementary Information.

## Data Availability

Data used in this study come from previously published sources. Data are available through the article of Kokkalas and Koukouvelas^9^—https://doi.org/10.1016/j.jog.2005.07.006.
