# Supplementary material for: Revealing the hidden signature of fault slip history in the morphology of degrading scarps
Source: Sci Rep. 2023 Mar 8;13:3856. doi: 10.1038/s41598-023-30772-z (PMC9995469; doi:10.1038/s41598-023-30772-z)
Supplement: Supplementary file 1 — Supplementary Information. [file 41598_2023_30772_MOESM1_ESM.pdf]

Revealing the hidden signature of fault slip history in the morphology of degrading scarps

List of authors:

Regina Holtmann[1,3,+], Rodolphe Cattin[2,*,+], Martine Simoes[1,+], and Philippe Steer[3,+]

[1] Université Paris Cité – CNRS, Institut de physique du globe de Paris, Paris, 75005, France.

[2] Université de Montpellier – CNRS, Géosciences Montpellier, Montpellier, 34000, France.

[3] Université de Rennes – CNRS, Géosciences Rennes, Rennes, 35000, France.

[*] email: rodolphe.cattin@umontpellier.fr

[+] these authors contributed equally to this work

Supplementary Information

Contents

Supplementary text S1 to S3
Supplementary Figure S1 to S5
References

Introduction

Supporting information gives (1) a detailed description of the approach based on a combination of diffused incremental topographies, (2) the Hanks et al.'s formulation[1] associated with the degradation of a scarp in the case of a creeping fault, (3) the analytical expression of the horizontal distance η_{max} associated with the maximal deviation, (4) a map of the Gulf of Corinth showing the location of the sites studied by Kokkalas and Koukouvelas[2], and (5) original raw satellite and field views used to generate Figure 1.

Text S1. A combination of diffused incremental topographies

As diffusion is uni-dimensional and linear, the profiles resulting from multiple events, can be constructed by extent through a combination of diffused incremental topographies. Based on equations (5) and (8), here we consider three different scenarios:

1. A single uplift scenario consisting of a single rupture (n=1) with uplift $u = u_{end}$ occurring at the beginning of the numerical experiment.
2. A two-uplift scenario, in which the fault experiences two (n=2) identical uplifts $u_{end}/2$ during the simulation, one at the beginning of the experiment and one at $t = t_{end}/2$.
3. A continuous uplift scenario associated with a creeping fault, in which the fault experiences a fractional uplift u_{end}/n at each iteration, with n the number of iterations. In our approach, we assume 10^4 time steps.

After the first time-step t_0, the three profiles significantly differ because they are associated with different vertical offsets (Figure S1a). The profile of a single event scenario is

$$\frac{z(x,t=t_0)}{u_{end}} = \frac{1}{2}\left[1+erf\left(x/\sqrt{4\kappa t_{end}}\right)\right], \qquad (S1)$$

whereas for a two-event it is

$$\frac{z(x,t=t_0)}{u_{end}} = \frac{1}{2\times 2}\left[1+erf\left(x/\sqrt{4\kappa t_{end}}\right)\right], \qquad (S2)$$

and for a continuous uplift

$$\frac{z(x,t=t_0)}{u_{end}} = \frac{1}{2\times n}\left[1+erf\left(x/\sqrt{4\kappa t_{end}}\right)\right]. \qquad (S3)$$

Just before the second event, while the vertical offsets associated with the two-events scenario and the creeping fault reach $u_{end}/2$, the two profiles show significant differences (Figure S1b). Due to the dimensionless approach, equation (S2) is still valid for the two-events scenario, whereas the profile associated with the creeping fault is

$$\frac{z(x,t=t_{end}/2)}{u_{end}} = \frac{1}{2\times n}\sum_{i=1}^{n/2}\left[1+erf\left(x/\sqrt{4\kappa t_{end}\times\frac{i}{n}}\right)\right]. \qquad (S4)$$

The second uplift in the two-event scenario creates an additional scarp, which degrades for a time-lapse $t_{end} - t_{end}/2$. The final profiles significantly differ from each other (Figure S1d). The greater the number of events, the steeper the slope near the fault.

Text S2. Hanks et al.'s formulation associated with a scarp degradation of a creeping fault

In the case of a creeping fault, the uplift rate can be viewed as a source term in the diffusion equation:

$$\frac{\partial z}{\partial t} = \kappa\frac{\partial^2 z}{\partial x^2} + A, \qquad (S5)$$

where $A = \frac{u_{end}}{t_{end}}$. Following Carslaw & Jaeger[3], Hanks et al.[1] propose the solution of equation (S5) for an intial scarp of offset $\pm a$ at $x \gtrless 0$, cutting a surface of slope b:

$$z(x,t) = (a+At)erf\left[\frac{x}{\sqrt{4\kappa t}}\right] + \frac{Ax^2}{2\kappa}\left\{erf\left[\frac{x}{\sqrt{4\kappa t}}\right] - sgn(x)\right\} + \frac{Ax}{\kappa}\sqrt{\frac{\kappa t}{\pi}}e^{-x^2/4\kappa t} + bx, \qquad (S6)$$

where $sgn(x \gtrless 0) = \pm 1$. In our approach $a = 0$ and $b = 0$, and the vertical offset only affects the block $x \geq 0$.

Equation (S6) becomes

$$\frac{z(x,t=t_{end})}{u_{end}} = \frac{1}{2}erf[\eta] + \eta^2\{erf[\eta] - sgn(\eta)\} + \frac{\eta}{\sqrt{\pi}}e^{-\eta^2} + \frac{1}{2}\,, \qquad (S7)$$

with $\eta = \frac{x}{\sqrt{4\kappa t_{end}}}$. This equation is used to validate our approach for a creeping fault (Figure 2a).

Text S3. Analytical expression of η_{max}

The maximum deviation between a multiple-events scenario and a single-event scenario is obtained for $\eta = \eta_{max}$. The expression of this parameter can be found from equations 6 and 8. Figure 2 shows this deviation, which is

$$\text{deviation} = \left(\frac{z(x,t_{end})}{u_{end}}\right)_n - \left(\frac{z(x,t_{end})}{u_{end}}\right)_1 \qquad (S8)$$

$$= \frac{1}{2\times n}\sum_{i=1}^{n}\left[1 + erf\left(x/\sqrt{4\kappa t_{end}\times\frac{i}{n}}\right)\right] - \left[1 + erf\left(x/\sqrt{4\kappa t_{end}}\right)\right]/2 \qquad (S9)$$

$$= \frac{1}{2\times n}\sum_{i=1}^{n}\left[1 + erf\left(\eta\times\sqrt{\frac{n}{i}}\right)\right] - [1 + erf(\eta)]/2\,. \qquad (S10)$$

The derivative of this deviation is then

$$\frac{d}{dx}(\text{deviation}) = \frac{1}{n}\frac{1}{\sqrt{\pi}}\sum_{i=1}^{n}\left[\sqrt{\frac{n}{i}}e^{-\frac{n}{i}\eta^2}\right] - \frac{1}{\sqrt{\pi}}e^{-\eta^2}\,. \qquad (S11)$$

For $\eta = \eta_{max}$ the derivative equals zero. η_{max} is then the solution of equation:

$$\frac{1}{n}\sum_{i=1}^{n}\left[\sqrt{\frac{n}{i}}e^{-\frac{n}{i}\eta^2}\right] - e^{-\eta^2} = 0. \qquad (S12)$$

For instance, for $n = 2$,

$$\frac{1}{2}\left[\sqrt{2}e^{-2\eta^2} + e^{-\eta^2}\right] - e^{-\eta^2} = 0 \Leftrightarrow e^{-\eta^2}\left(\sqrt{2}e^{-\eta^2} - 1\right) = 0$$

$$\Leftrightarrow \eta = \pm\eta_{max} = \pm\sqrt{\left(ln\left(\sqrt{2}\right)\right)} \approx \pm 0.6. \qquad (S13)$$

Figure S1.

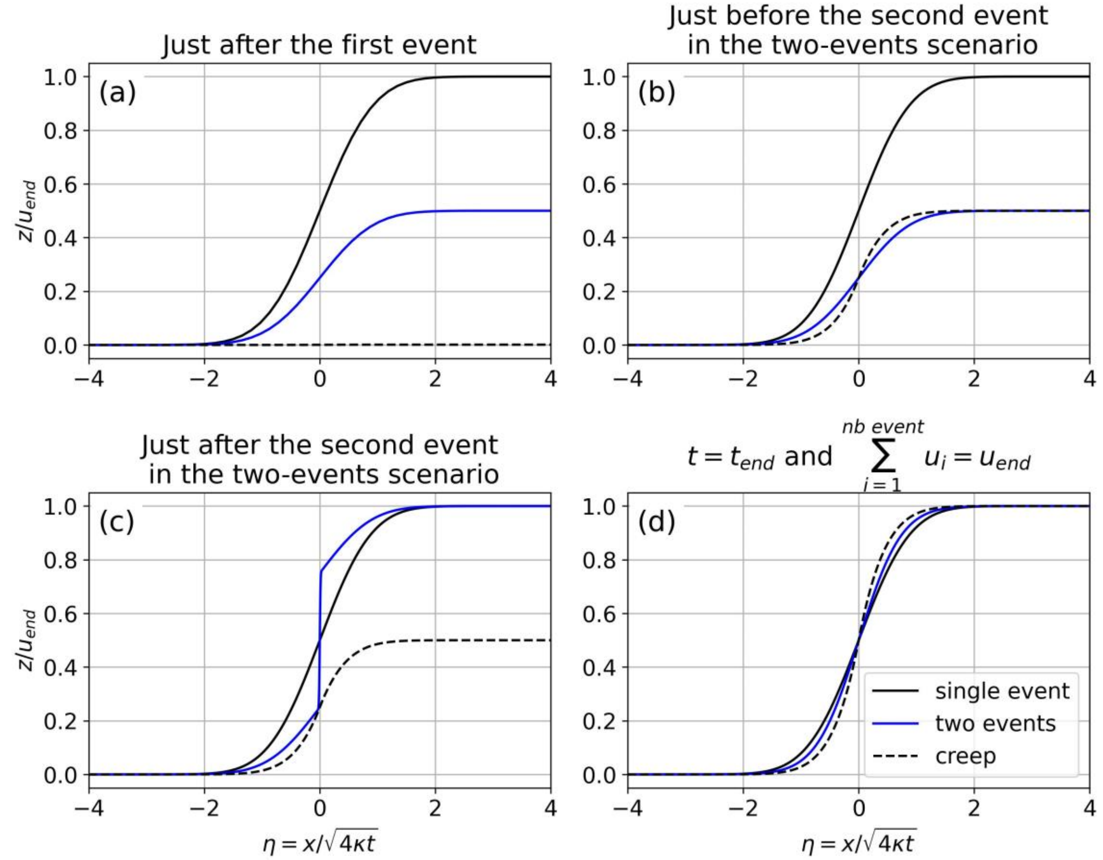

Figure S1. Fault scarp degradation associated with three different fault slip histories, in which the total uplift u_{end} is reached after either a single uplift event, two events or by creep, over a total time-lapse t_{end}. (a) Dimensionless elevation profiles after one time step, i.e., $t_{end}/10^4$ in our approach. Black, blue, and dashed lines are associated with single-event, two-events, and creeping fault scenarios, respectively. (b) Same as (a) but at time $t_{end}/2$, just before the occurrence of the second earthquake in the two-events scenario. (c) Profiles just after the occurrence of the second earthquake. (d) Final profiles obtained after a time-lapse t_{end} when the cumulative uplift reaches u_{end}. Note that the average slope of the scarp is correlated to the number of events.

Figure S2.

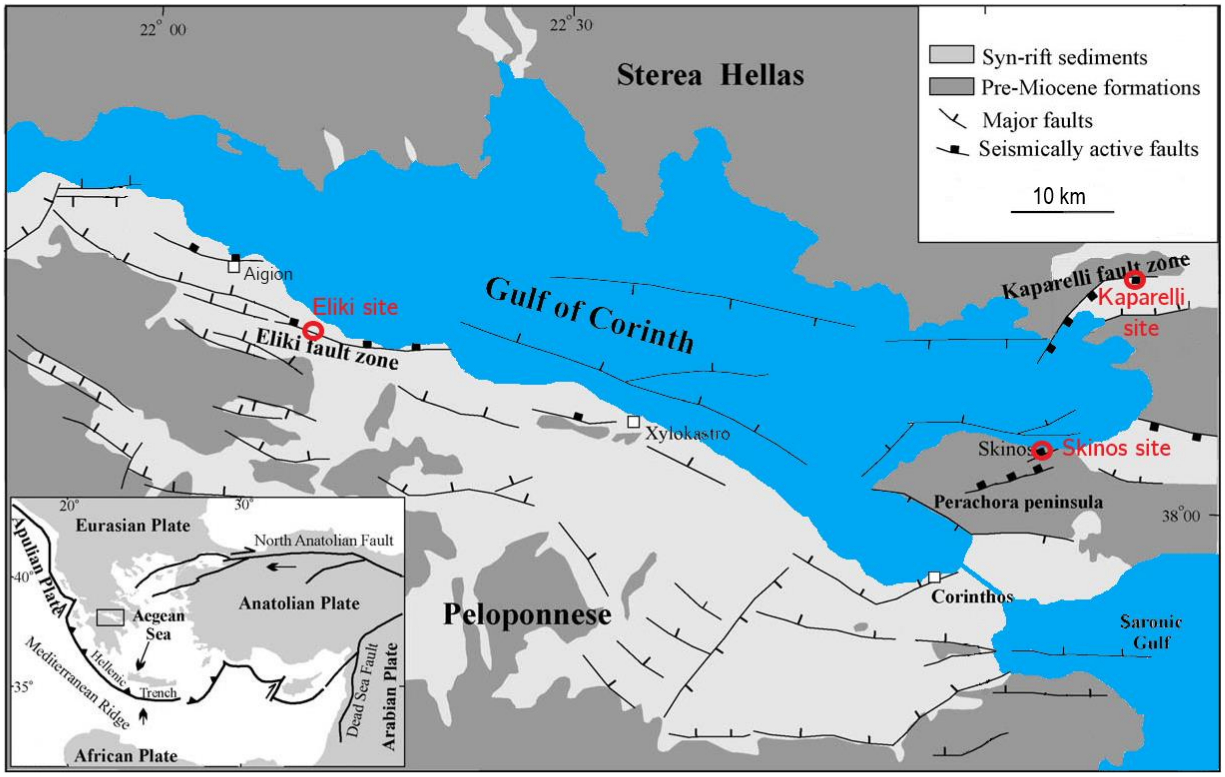

Figure S2. Simplified map of the Gulf of Corinth showing the location of the major faults (modified from Kokkalas and Koukouvelas[2]). Red circles give the location of the Eliki, Kaparelli and Skinos sites studied by Kokkalas and Koukouvelas[2]. Only the estimated diffusion coefficients from the Eliki site are used in this study. Inset shows the associated geodynamical setting and the location of the Gulf of Corinth (black rectangle).

Figure S3.

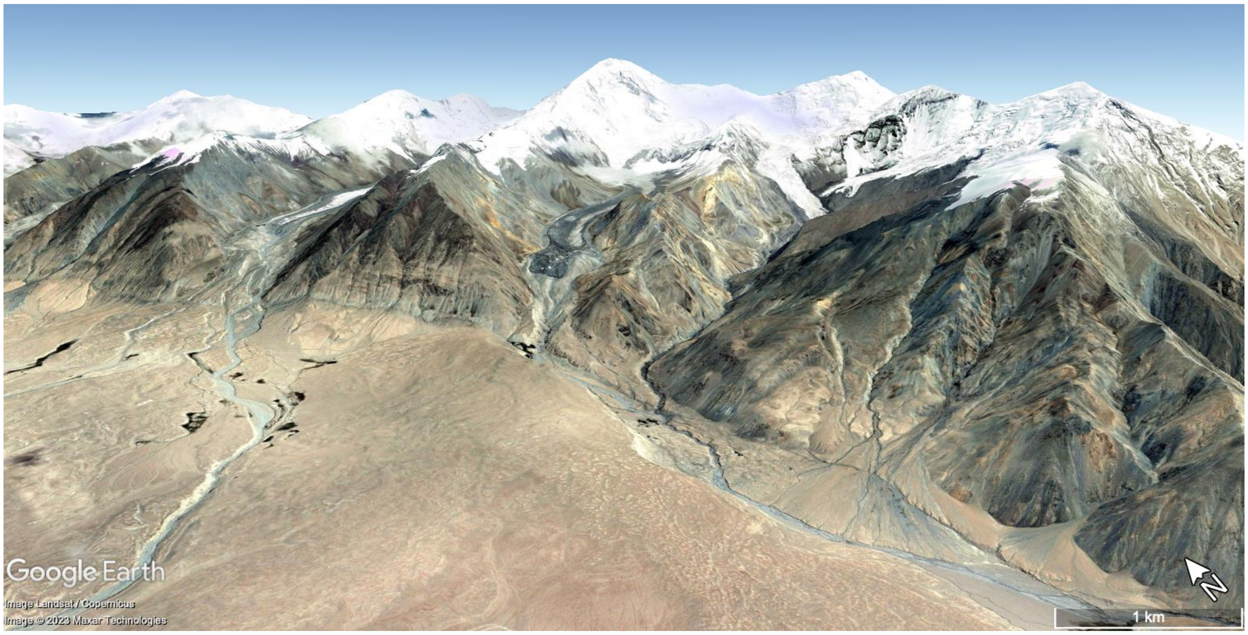

Figure S3. Satellite view taken from the Google Earth database to generate Figure 1. © Google Earth (2023 Maxar Technologies and Landsat / Copernicus), satellite image taken on July 9th 2007. View from the road G314 from Kashgar to Tashgorgan (38° 43' 42.97" N, 75° 0' 59.51" E).

Figure S4.

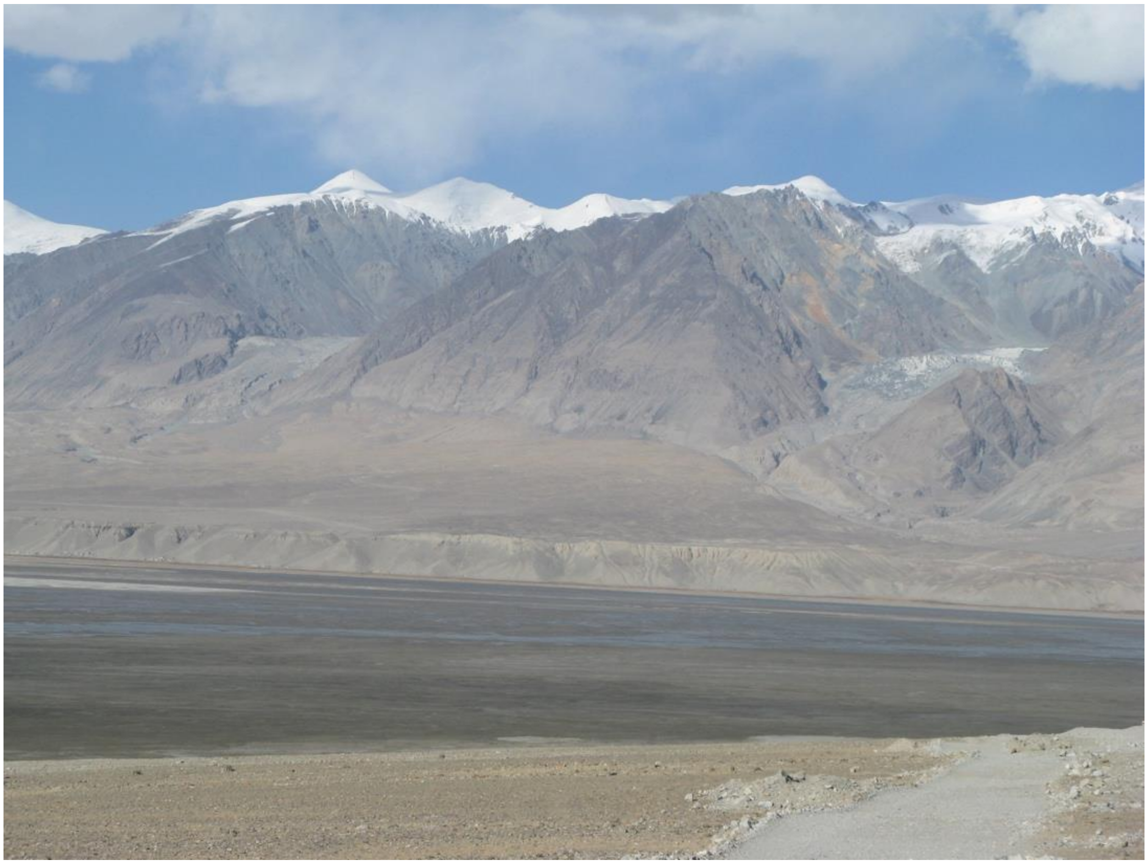

Figure S4: Original field picture, used to generate the lower left field view of Figure 1. Picture taken from the road G314 from Kashgar to Tashgorgan (38° 43' 42.97" N, 75° 0' 59.51" E) on October 18th 2007. The lower left field picture of Figure 1 only shows a detailed view of the fault scarp visible in this field picture (upper left corner of this picture). The contrast of this detailed view has been enhanced using Adobe Photoshop to generate the inset posted in Figure 1.

Figure S5.

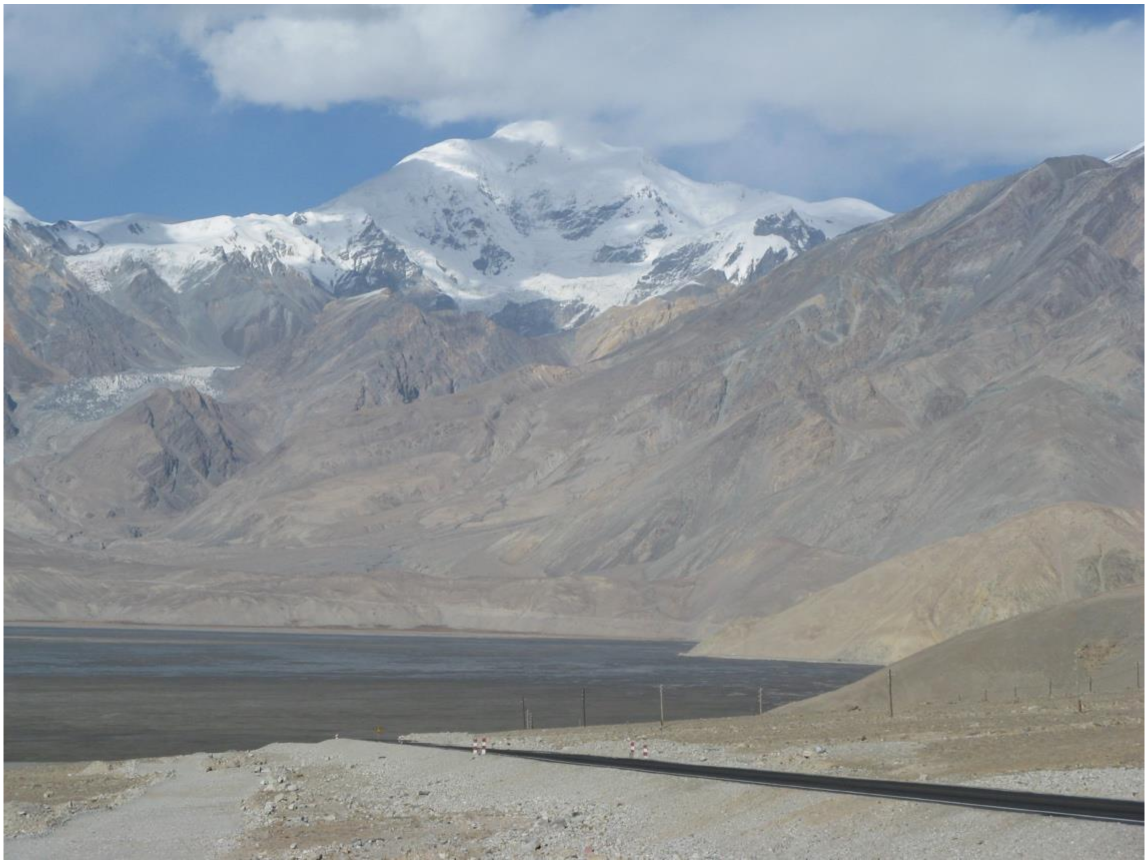

Figure S5: Original field picture, used to generate the lower right field view of Figure 1. Picture taken from the road G314 from Kashgar to Tashgorgan (38° 43' 42.97" N, 75° 0' 59.51" E) on October 18th 2007. The lower right field picture of Figure 1 only shows a detailed view of the fault scarp visible in this field picture, where the fault disrupts an alluvial fan (at the foot of the mountain front, in the lower center of the picture). The contrast of this detailed view has been enhanced using Adobe Photoshop to generate the inset posted in Figure 1.

References

1. Hanks, T. C., Bucknam, R. C., Lajoie, K. R. & Wallace, R. E. Modification of wave-cut and faulting-controlled landforms. *J. Geophys. Res.: Solid Earth* 89(B7), 5771–5790, DOI: https://doi.org/10.1029/JB089iB07p05771 (1984).
2. Kokkalas, S. & Koukouvelas, I. K. Fault-scarp degradation modeling in central Greece: the Kaparelli and Eliki faults (Gulf of Corinth) as a case study. J. Geodyn. 40(2-3), 200–215, DOI: https://doi.org/10.1016/j.jog.2005.07.006 (2005).
3. Carslaw, H.S. & Jaeger, J.C. *Conduction of heat in solids.* 79 (Oxford University Press, 1959).
